# INSOMNIA SYMPTOM TRAJECTORIES OF SPOUSE CAREGIVERS OF OLDER ADULTS WITH FUNCTIONAL LIMITATIONS

**DOI:** 10.1093/geroni/igac059.3114

**Published:** 2022-12-20

**Authors:** Dexia Kong, Peiyi Lu

**Affiliations:** The Chinese University of Hong Kong, Hong Kong, Hong Kong; Columbia University, New York, New York, United States

## Abstract

**Background:**

Studies have suggested older spouse caregivers experience burden-related adverse health outcomes compared to non-caregivers. However, potential causal inferences remain unclear. This study examined the effect of caregiving on insomnia symptoms of spouse caregivers over time, compared to non-caregiver samples matched by propensity score (PS).

**Methods:**

Longitudinal data from the Health and Retirement Study from 2006 to 2018 were used. Caregivers (Nf403) were respondents (aged 50+) who assisted their heterosexual spouses in performing (instrumental) activities of daily living at baseline. PS-matching was used to match non-caregivers based on sociodemographic, household, and health-related characteristics. Symptoms of insomnia were evaluated every four years for both groups. The link between caregiver status and number/severity of insomnia symptoms over time was assessed using a Poisson mixed-effects model.

**Results:**

The propensity-matched sample achieved a satisfactory covariate balance. There was no statistically significant difference between caregivers and non-caregivers in the number of insomnia symptoms at baseline (β_caregiver=0.092, 95% CI = -0.017, 0.201). However, caregivers reported a slower increase in insomnia symptoms compared to non-caregivers (β_caregiver×time = -0.012, 95% CI = -0.021, -0.003). Results were cross-validated in modelling the severity of insomnia symptoms.

**Conclusion:**

There is weak evidence that a spouse’s role as a caregiver may be beneficial for his/her sleep health over time. The negative effects of caring on older individuals’ sleep may vary depending on the caregiving context. The potential health benefits of informal spousal caring and their underlying mechanisms warrant further investigations.

